# Influence of Mesenchymal Stem Cell Sources on Their Regenerative Capacities on Different Surfaces

**DOI:** 10.3390/cells10020481

**Published:** 2021-02-23

**Authors:** Arkaitz Mucientes, Eva Herranz, Enrique Moro, Aranzazu González-Corchón, María Jesús Peña-Soria, Lydia Abasolo, Luis Rodriguez-Rodriguez, Jose Ramon Lamas, Benjamín Fernández-Gutiérrez

**Affiliations:** 1 UGC de Reumatología, Hospital Clínico San Carlos, IdISSC, 28040 Madrid, Spain; arkaitz.mucientes@salud.madrid.org (A.M.); evaherranzdlp@hotmail.com (E.H.); lydia.abasolo@salud.madrid.org (L.A.); lrrodriguez@salud.madrid.org (L.R.-R.); jrlamas@gmail.com (J.R.L.); 2 UGC de Traumatología, Hospital Clínico San Carlos, IdISSC, 28040 Madrid, Spain; lmoro@ucm.es; 3 Servicio de Cirugía Oral y Maxilofacial, Hospital Clínico San Carlos, IdISSC, 28040 Madrid, Spain; agcorchon@yahoo.es; 4 Servicio de Cirugía I, Hospital Clínico San Carlos, IdISSC, 28040 Madrid, Spain; mariajesus_penasoria@hotmail.com

**Keywords:** mesenchymal stem cell, dental pulp, beta-tricalcium phosphate, hydroxyapatite, osteogenesis, regenerative medicine

## Abstract

Current gold-standard strategies for bone regeneration do not achieve the optimal recovery of bone biomechanical properties. To bypass these limitations, tissue engineering techniques based on hybrid materials made up of osteoprogenitor cells—such as mesenchymal stem cells (MSCs)—and bioactive ceramic scaffolds—such as calcium phosphate-based (CaPs) bioceramics—seem promising. The biological properties of MSCs are influenced by the tissue source. This study aims to define the optimal MSC source and construct (i.e., the MSC–CaP combination) for clinical application in bone regeneration. A previous iTRAQ analysis generated the hypothesis that anatomical proximity to bone has a direct effect on MSC phenotype. MSCs were isolated from adipose tissue, bone marrow, and dental pulp, then cultured both on a plastic surface and on CaPs (hydroxyapatite and β-tricalcium phosphate), to compare their biological features. On plastic, MSCs isolated from dental pulp (DPSCs) presented the highest proliferation capacity and the greatest osteogenic potential. On both CaPs, DPSCs demonstrated the greatest capacity to colonise the bioceramics. Furthermore, the results demonstrated a trend that DPSCs had the most robust increase in ALP activity. Regarding CaPs, β-tricalcium phosphate obtained the best viability results, while hydroxyapatite had the highest ALP activity values. Therefore, we propose DPSCs as suitable MSCs for cell-based bone regeneration strategies.

## 1. Introduction

Bone regeneration and bone remodelling can be considered two sides of the same coin. While bone remodelling is a life-long process, bone regeneration occurs mainly during bone healing [1]. It is described that, for small fractures or injuries, the bone has an innate ability to repair without scarring [2]. These regenerative processes are largely surpassed by major bone damage, such as skeletal reconstruction surgeries, bone defects of various origins (e.g., traumatic, infectious, or tumoral), or congenital skeletal dysplasias. Current orthopaedic surgery strategies are mostly bone grafting (both autologous and allogeneic) and osteodistraction [3]. They can be combined with the use of growth factors or osteoinductive scaffolds. However, the successful recovery of the biomechanical properties of bone is limited. To bypass these limitations, novel strategies have been developed. Among these, hybrid materials consisting of osteoprogenitor cells, such mesenchymal stem cells (MSCs) and bioactive ceramic scaffolds, have been proposed as promising tools [4].

Calcium phosphate-based (CaPs) bioceramics have been used both in maxillo-facial surgeries and dentistry for over 30 years. CaPs are biocompatible, osteoconductive, osteoinductive and, in some cases, bioactive. Due to these properties, CaPs have been widely used as scaffolds for bone regeneration and tissue engineering. Among the different CaPs bioceramics, hydroxyapatite (HA) and β-tricalcium phosphate (β-TCP) have been widely used [5]. The Ca:P ratio determines certain properties, such as solubility and tendency for resorption in the body [6]. HA is considered relatively non-biodegradable, while β-TCP degrades readily and can be completely replaced by newly formed bone.

Adult MSCs can be isolated from almost every tissue [7]. They have multipotential capacities and can differentiate into cells of several mesenchymal lineages (e.g., cartilage, bone, tendons, muscles, and adipocytes), among other cell types [8]. Moreover, MSCs have migratory and homing abilities that are crucial for wound healing, and their immunomodulatory and immunological tolerance induction potentials have been communicated [9,10,11]. Although MSCs derived from different sources present similar phenotypic characteristics, the intensity of these varies depends on the tissue source [12].

Bone marrow MSCs (BM-MSCs) were the first MSCs known, described by Friedenstein et al. [13]. They have been widely used and studied in trials looking for potential regenerative therapies. Adipose-derived MSCs (ASCs) present several advantages, compared to MSCs obtained from other sources: Adipose depots are ubiquitous in the body, easily accessible with minimal invasion, and contain a large number of stem cells [14]. Furthermore, their capacity to promote osteogenesis in animal models has been described [15]. MSCs isolated from dental pulp (DPSCs) are usually isolated after the surgical removal of wisdom teeth and, so, they are considered a non-invasive source of MSCs. DPSCs present a high proliferative capacity and easily differentiate into odontoblasts, osteoblasts, and chondrocytes [16,17]. DPSCs have been recently proposed for use in regenerative therapies for bone diseases, among other conditions [18].

Considering the osteogenic potential as a property influenced by the tissue, several comparative studies of MSCs derived from various sources on different surfaces have been published [19,20,21,22,23,24]. Despite these studies, clarification is still needed regarding the influence of the cell source and surface on the osteogenic potential. Osteogenic potential maximisation in the context of bone-regeneration processes would serve as an important step. This study aims to define the MSC source and construct (MSC and scaffold) combination with the highest osteogenic potential.

## 2. Materials and Methods

### 2.1. Proteomic Analysis by iTRAQ Labelling

Data were obtained in a previous proteomic analysis, in which MSCs from subchondral bone and cartilage were compared (not published). Specifically, the results obtained after comparing these locations in healthy individuals were analysed.

Briefly, MSCs were obtained surgically from subchondral bone and cartilage of a donor without osteoporotic or osteoarthritic signs. Total protein content was isolated, dried in air, and then re-suspended in 25 μL of iTRAQ dissolution buffer (ABSciex, Foster City, CA, USA). Protein concentrations were determined by Bradford assay (Sigma-Aldrich, St. Louis, MO, USA). TRAQ labelling (ABSciex, Foster City, CA, USA) was performed with SB(116) and C(117) mass tags and desalted with home-made C-18 Stage-tips. Fractions separated in a nanoLC system (Tempo, Eksigent) were automatically deposited on a MALDI plate and analysed by MS/MS (4800 MALDI-TOF/TOF system; ABSciex). Relative quantitative analysis was performed using the ProteinPilot software (ABSciex) with the Paragon™ Algorithm for protein identification and quantification. Only proteins identified with at least 95% confidence, a Prot Score of at least 1.3, a *p*-value ≤ 0.05, and ratio ≠ 1 were considered as modulated.

### 2.2. Samples

MSCs from three different localisations (bone marrow, dental pulp, and adipose tissue), obtained from healthy donors (Table 1), were used for in vitro biological studies. Written informed consent was obtained from all donors before sample collection. The study was approved following the guidelines of the institutional ethics committee (Comité Ético de Investigación Clínica Hospital Clínico San Carlos) and the principles expressed in the Declaration of Helsinki.

Discs of CaPs bioceramics used in this study were synthesised, sintered, and polished (if needed). They were kindly supplied by the Instituto de Cerámica y Vidrio (ICV-CSIC). The discs had a diameter of 18 mm and 4 mm thickness. The sintering temperature employed for β-TCP was 1130 °C, and that for HA was 1250 °C [25,26].

### 2.3. Cell Isolation and Culture

ASCs were obtained from adipose tissue after surgical biopsies, according to Yang et al. [27]. DPSCs were isolated after dental pulp mechanical extraction from wisdom exodontias, as described by Huang et al. [28]. Finally, BM-MSCs were obtained from femoral channel aspirates of bone marrow, taken during joint replacement surgery, in a Ficoll density gradient and cultured directly, as described by Gudlevicine et al. [29].

Once isolated, cells were expanded in growth medium: DMEM supplemented with 10% FBS and antibiotics. DPSCs required 20% FBS, instead of the 10% usually established, as described by Alkhalil et al. [30]. Cell cultures were expanded at 37 °C in a 5% CO_2_ atmosphere. The medium was changed every 3 days until cell confluence at passage 3.

### 2.4. Cell Characterisation

In order to confirm that cells satisfied the minimal criteria for the definition of MSC proposed by the International Society for Cellular Therapy [31], flow cytometry and histochemistry assays were carried out, as we have previously described [32].

### 2.5. Biological Features on the Plastic Surface

#### 2.5.1. MSC Proliferation Rate

During cell expansion, cell proliferation was evaluated by calculating the population doubling time (Dt), which is defined by:

Dt = T ln2/ln(Nf/Ni)

where “T” represents the time elapsed between determinations of the final number of cells (Nf) obtained from an initial cell number (Ni).

#### 2.5.2. Osteogenic Commitment of Each MSC

Alizarin Red staining was carried out along culture on plastic. The coloured area of cell cultures after Alizarin red staining was quantified, in terms of percentage, using the ImageJ 1.43v software (National Institutes of Health, freely available: https://imagej.nih.gov/ij/index.html, accessed on 5 November 2020).

### 2.6. Cell Behaviour on CaPs

#### 2.6.1. Cell Activity/Viability

Prior to cell seeding, the scaffolds were previously submerged in growth medium for 24 h, then seeded with 1 mL of a cellular suspension containing 50,000 cells.

Monitoring of cell viability was conducted using the colourimetric indicator AlamarBlue™ (Cat#Y00-100. Thermo Fisher Scientific, Waltham, MA USA). The variation of absorbance at 570 nm was measured at 24 h, 4 days, and 7 days using a Heales MB-580 microplate reader (Shenzhen Heales Technology Development Co. Ltd., Shenzhen, China). The amount of absorbance corresponded to cell metabolic activity.

#### 2.6.2. Scanning Electronic Microscopy (SEM)

The cellular organisation, adhesion, and colonisation of the scaffolds were assessed at 24 h and 7 days. Each sample was subjected to fixation with phosphate buffer solution containing 4% paraformaldehyde and 2.5% glutaraldehyde for 30 min. After fixation, samples were washed 3 times with phosphate-buffered saline (PBS) for 20 min, followed by incubation for 45 min with a solution of 1% osmium tetraoxide and, finally, washed again with PBS 3 times for 10 min. The next step was the dehydration of samples by immersing them in increasing ethanol concentrations: 30%, 50%, 70%, 96%, and 100%. The final step was to introduce the samples into a critical-point device and cover them with vaporised gold. Samples were then observed and analysed by scanning electron microscopy (SEM JEM 6400, JEOL, Akishima-shi, Japan).

#### 2.6.3. ALP Activity

Early osteoblast differentiation was evaluated by measuring the alkaline phosphatase (ALP) activity, which is expressed just before the matrix mineralisation occurs, and its role as an osteogenic activity marker is established [33]. The evaluation was made using 24-well plates, and three conditions for each sample were evaluated: cell in plastic with growth medium as an internal control, and cells seeded on HA with osteogenic medium or β-TCP with osteogenic medium. Duplicates for each experimental condition were made.

Osteogenesis progression was measured between 24 h and 7 days. At these time points, media were discarded, and ceramic discs recovered, washed with PBS, stored at −20 °C, and soaked in lysis buffer (0.1 wt%, Triton-X 100, 1 mM MgCl_2_, 0.1 mM ZnCl_2_). ALP activity was determined by a colourimetric method using a commercial kit (Thermo Fisher Scientific, Cat#37629), following the manufacturer instructions, and measuring the absorbance at 405 nm.

### 2.7. Statistical Analysis

Statistical analysis was performed using the GraphPad Prism version 7.00 software for Windows (GraphPad Software, La Jolla, CA, USA; www.graphpad.com, accessed on 5 November 2020). We used one-way ANOVA followed by Bonferroni post-hoc tests and two-tailed paired/unpaired Student t-tests for comparison of normal variables. The level of significance *p* < 0.05 was considered statistically significant. Each experiment was performed with replicates.

## 3. Results

### 3.1. iTRAQ Results Analysis

The difference in protein components between MSCs from subchondral bone and cartilage were analysed using an iTRAQ-based comparative analysis. The results obtained revealed the identification of 1012 unique proteins in the samples. Fifty of these proteins displayed statistically significant differences (Table 2). Among those, five proteins have been previously associated with the osteoblast differentiation process: PALLD, HSPA5/GRP78, FLNA, IGFBP3, and DSTN.

### 3.2. Biological Features on the Plastic Surface

**Figure 1:**
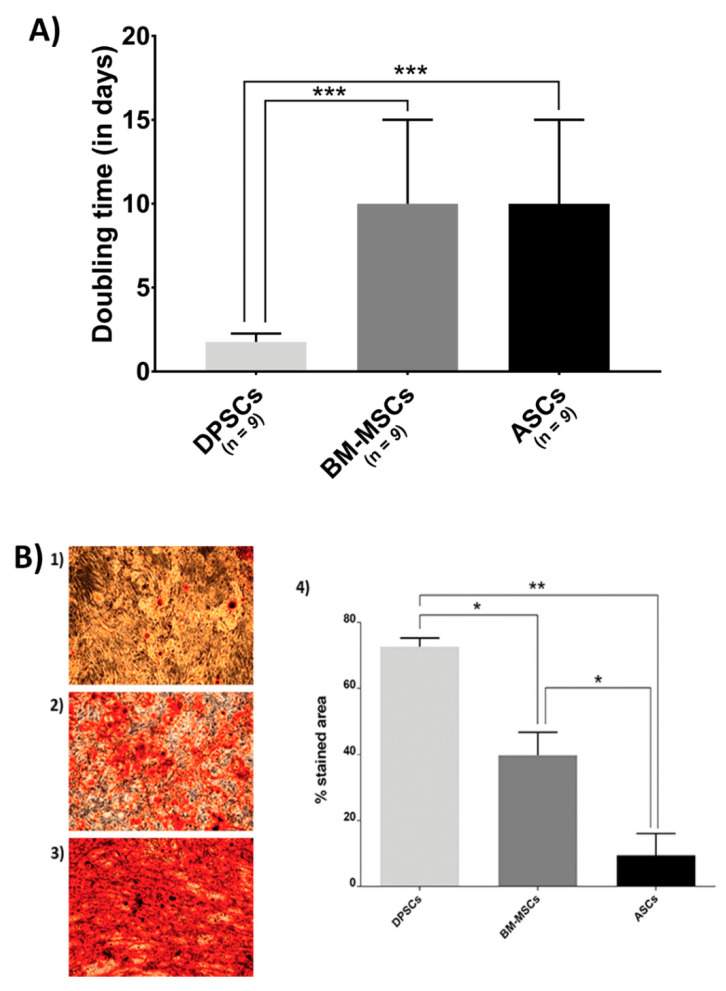
Biological features of MSCs on the plastic surface: (**A**) MSC proliferation. Doubling time (in days) when cultured in plastic presented by MSC isolated from adipose tissue (ASCs), dental pulp (DPSCs), and bone marrow (BM-MSCs); and (**B**) Alizarin Red staining and its quantification of different source-derived mesenchymal stem cells—(1) Alizarin Red staining of ASCs after 21 days of culture in osteogenic medium (×10); (2) Alizarin Red staining of BM-MSCs after 21 days of culture in osteogenic medium (×10); (3) Alizarin Red staining of DPSCs after 21 days of culture in osteogenic medium (×10); and (4) Alizarin Red staining quantification of MSC, according to the cell source, adipose tissue (ASCs), dental pulp (DPSCs), and bone marrow (BM-MSCs). All data are shown as mean ± standard deviation. Significance level: * *p* ≤ 0.05, ** *p* ≤ 0.01 *** *p* ≤ 0.001.

The proliferation rate and osteogenic potential of MSCs when cultured on plastic surfaces were measured (Figure 1).

Figure 1A shows the proliferation rate results. BM-MSCs and ASCs showed identical doubling time, while DPSCs proliferated more rapidly (ASCs = 10 days, BM-MSCs = 10 days, DPSCs = 1.76 days, *p*_ANOVA_ < 0.0001, *p*_DPSC-ASC_ = 0.0003, *p*_DPSC-BM-MSC_ = 0.0003, *p*_ASC-BM-MSC_ > 0.9999).

Furthermore, Alizarin Red staining showed that DPSCs presented a much more extended stained area than the other sources (Figure 1B), where its quantification ratified the significant differences among MSCs (ASCs = 9.4210%, BM-MSCs = 39.7150%, DPSCs = 72.5965%; *p*_ANOVA_ = 0.0038, *p*_DPSC-ASC_ = 0.0063, *p*_DPSC-BM-MSC_ = 0.0250, *p*_ASC-BM-MSC_ = 0.0473).

### 3.3. Cell Behaviour on CaPs

#### 3.3.1. Viability Test

**Figure 2:**
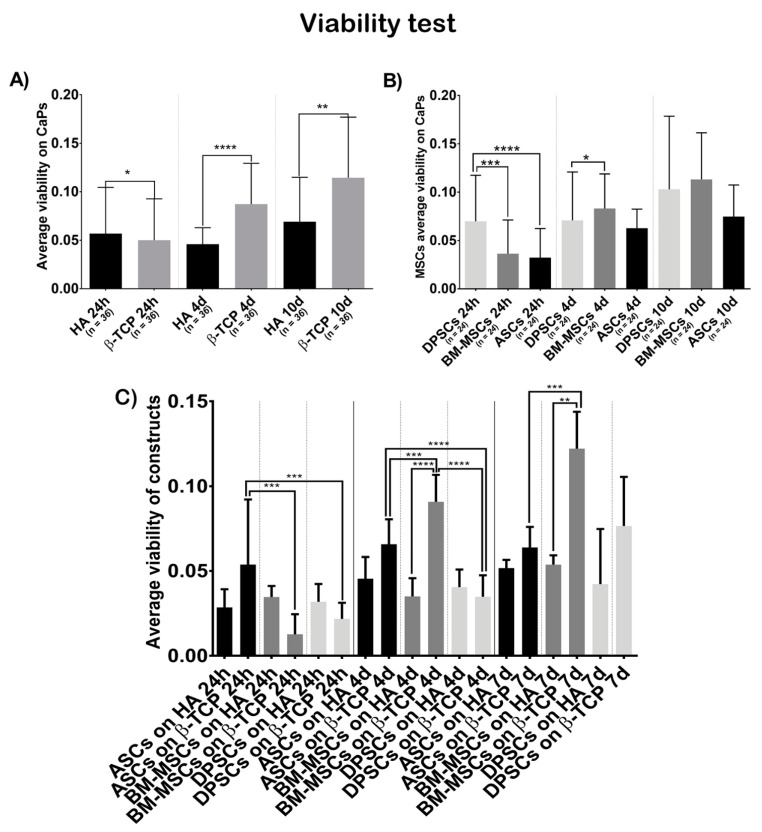
Cell viability, in terms of optical density at 570 nm, when seeded on CaPs: (**A**) average viability shown by all MSCs on each CaP—Hydroxyapatite (HA) and ß-tricalcium phosphate (β-TCP); (**B**) average viability shown by each MSC on both CaPs—Adipose tissue (ASCs), dental pulp (DPSCs), and bone marrow (BM-MSCs); (**C**) average viability shown by each construct. All data are shown as mean ± standard deviation. Significance level: * *p* ≤ 0.05, ** *p* ≤ 0.01, *** *p* ≤ 0.001, **** *p* ≤ 0.0001.

Figure 2A shows that cells on β-TCP had higher viability values than cells on HA (*t* = 24 h, β-TCP = 0.0500, HA = 0.0568, *p* = 0.0246; *t* = 4 days, β-TCP = 0.0872, HA = 0.0459, *p* ˂ 0.0001; *t* = 7 days, β-TCP = 0.1144, HA = 0.0690, *p* = 0.0021).

Figure 2B shows that the viability test results were not different, regarding the cell source at day 7 (*t* = 7 days, ASCs = 0.0748, BM-MSCs = 0.1131, DPSCs = 0.1029, *p*_ANOVA_ = 0.2141, *p*_DPSC-ASC_ > 0.9999, *p*_DPSC-BM-MSC_ = 0.0206, *p*_ASC-BM-MSC_ = 0.2584), but there were significant differences favouring DPSCs at 24 h and at 4 days (*t* = 24 h, ASCs = 0.0323, BM-MSCs = 0.0363, DPSCs = 0.0699, *p*_ANOVA_ ˂ 0.0001, *p*_DPSC-ASC_ < 0.0001, *p*_DPSC-BM-MSC_ = 0.0003, *p*_ASC-BM-MSC_ > 0.9999; *t* = 4 days, ASCs = 0.0627, BM-MSCs = 0.0831, DPSCs = 0.0708, *p*_ANOVA_ = 0.0249, *p*_DPSC-ASC_ > 0.9999, *p*_DPSC-BM-MSC_ = 0.7430, *p*_ASC-BM-MSC_ = 0.2460). When analysing the viability of each construct (Figure 2C), BM-MSCs showed significant higher values seeded on β-TCP while ASCs and DPSCs did not (*t* = 24 h, ASCs + HA = 0.0285, ASCs + β-TCP = 0.05375, BM-MSCs + HA = 0.0346, BM-MSCs + β-TCP = 0.01267, DPSCs + HA = 0.0318, DPSCs + β-TCP = 0.02171, *p*_ANOVA_ = 0.0002; *t* = 4 days, ASCs + HA = 0.0454, ASCs + β-TCP = 0.0658, BM-MSCs + HA = 0.0350, BM-MSCs + β-TCP = 0.0908, DPSCs + HA = 0.0404, DPSCs + β-TCP = 0.0348, *p*_ANOVA_ < 0.0001; *t* = 7 days, ASCs + HA = 0.0517, ASCs + β-TCP = 0.0638, BM-MSCs + HA = 0.0537, BM-MSCs + β-TCP = 0.1222, DPSCs + HA = 0.0422, DPSCs + β-TCP = 0.0765, *p*_ANOVA_ = < 0.0001).

#### 3.3.2. Scanning Electronic Microscopy

Cell morphology and behaviour, when grown on β-TCP and HA scaffolds, were studied by SEM at both 24 h and 7 days after culture. In all cases, cells did not show visual signs of cytotoxicity. Cells were visualised in their normal shape and size.

**Figure 3:**
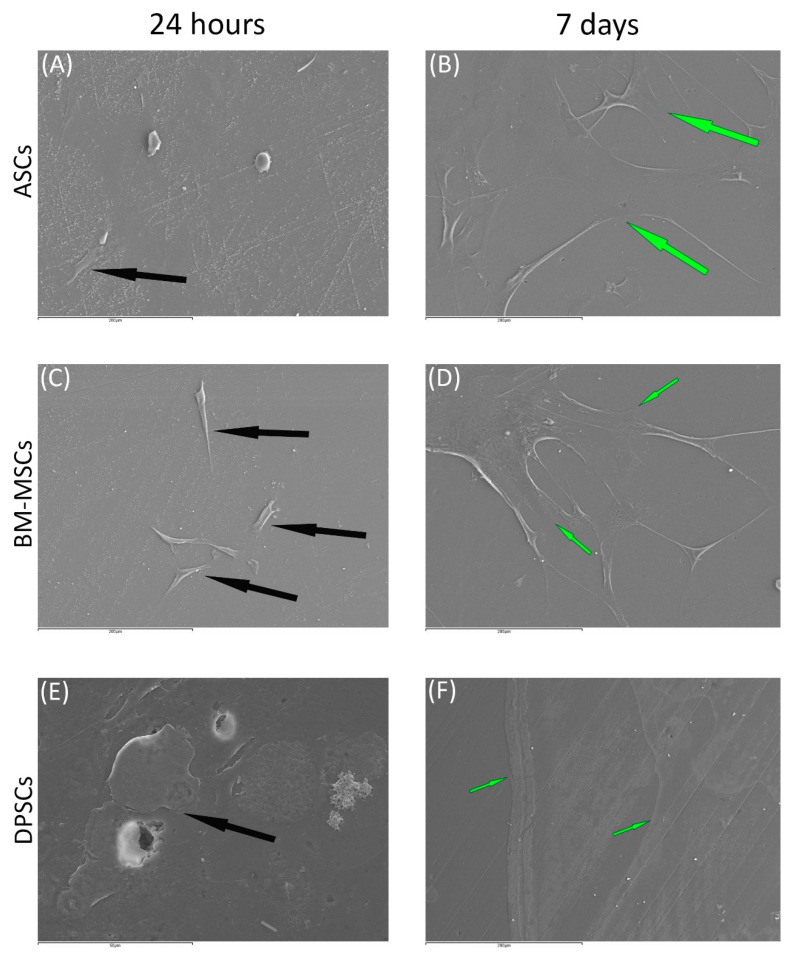
The behaviour of MSCs seeded on ß-TCP scaffold at different time points: (**A**) ASCs on ß-TCP scaffold after 24 h (scale bar: 200 μm); (**B**) ASCs forming cell layer on ß-TCP scaffold after 7 days (scale bar: 200 μm); (**C**) BM-MSCs on ß-TCP scaffold after 24 h (scale bar: 200 μm); (**D**) BM-MSCs forming cell layer on ß-TCP scaffold after 7 days (scale bar: 200 μm); (**E**) DPSCs on ß-TCP scaffold at 24 h (scale bar: 50 μm); and (**F**) DPSCs forming cell layer on ß-TCP scaffold after 7 days (scale bar: 200 μm). Black arrows indicate unique cells. Green arrows indicate cell layers.

**Figure 4:**
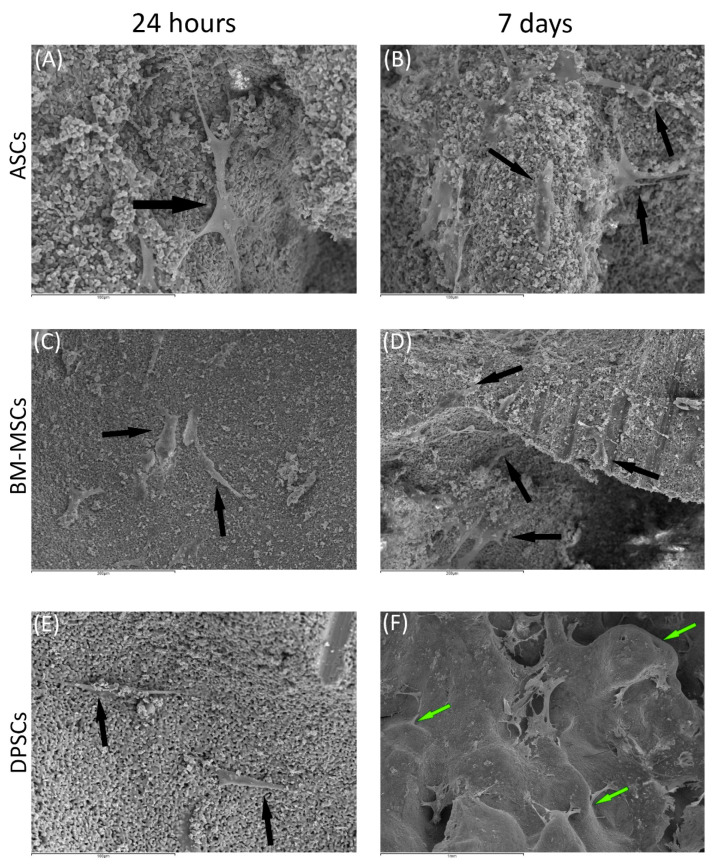
The behaviour of MSCs seeded on HA scaffold at different time points: (**A**) ASCs on HA scaffold after 24 h (scale bar: 100 μm); (**B**) ASCs forming cell layer on HA scaffold after 7 days (scale bar: 100 μm); (**C**) BM-MSCs on HA scaffold after 24 h (scale bar: 200 μm); (**D**) BM-MSCs forming cell layer on HA scaffold after 7 days (scale bar: 200 μm); (**E**) DPSCs on HA scaffold at 24 h (scale bar: 100 μm); and (**F**) DPSCs forming cell layer on HA scaffold after 7 days (scale bar: 1 mm). Black arrows indicate unique cells. Green arrows indicate cell layers.

**Figure 5:**
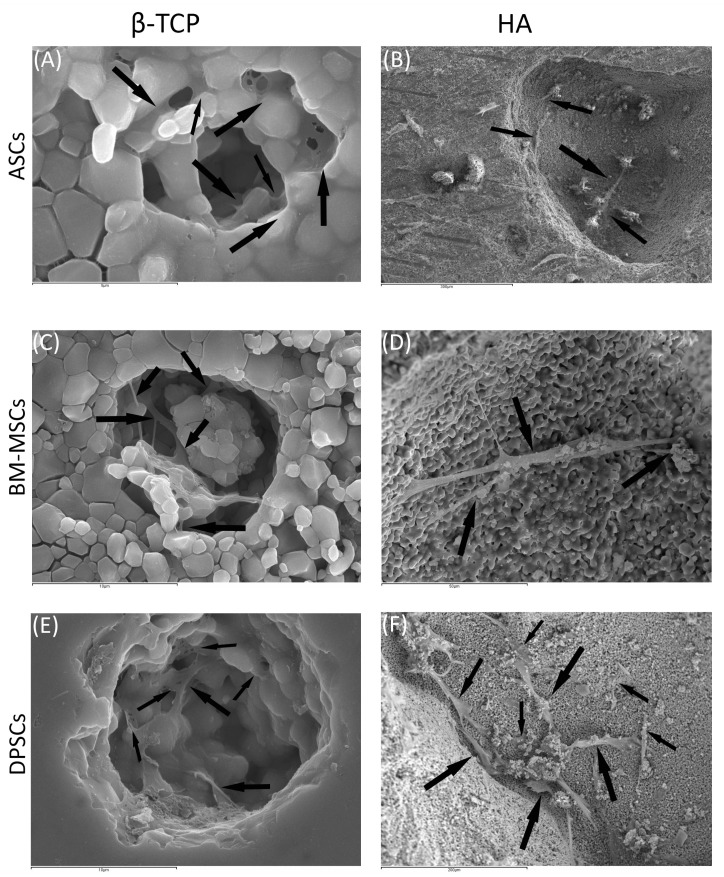
MSCs colonising pores of both HA and ß-TCP scaffolds after 24 h: (**A**) existing pores in HA colonised by DPSCs (scale bar: 5 μm); (**B**) existing pores in HA colonised by BM-MSCs (scale bar: 300 μm); (**C**) existing pores in HA colonised by ASCs (scale bar: 10 μm); (**D**) existing pores in ß-TCP colonised by DPSCs (scale bar: 50 μm); (**E**) existing pores in ß-TCP colonised by BM-MSCs (scale bar: 10μm); and (**F**) existing pores in ß-TCP colonised by ASCs (scale bar: 200 μm). Black arrows indicate unique cells.

Regarding β-TCP, cell adhesion seemed to be faster in BM-MSCs (Figure 3C) than in ASCs and DPSCs (Figure 3A,E, respectively). After 7 days, cell overlays were observed in β-TCP discs, independently of the MSC seeded (Figure 3B,D,F). When culturing on HA, the adhesion process was similar for all MSCs (Figure 4A,C,E). On day 7, BM-MSCs and ASCs (Figure 4B–D) exhibited similar appearances, where the number of cells increased without forming layers. In contrast, DPSCs (Figure 4F) formed a multilayer covering the entire surface. Finally, all MSCs colonised the existing internal pores in both CaPs (Figure 5).

#### 3.3.3. Osteogenic Potential

**Figure 6:**
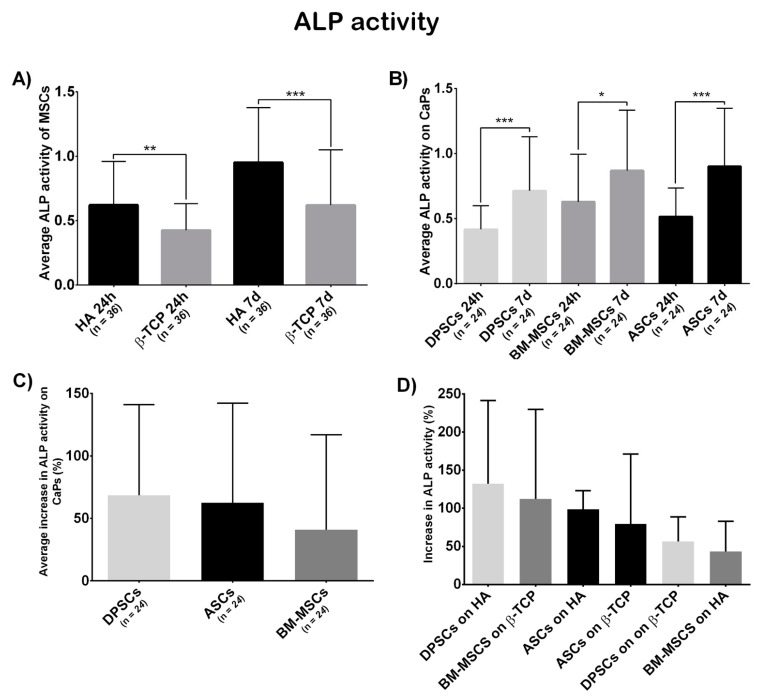
(**A**) Average ALP activity shown by all MSCs on each CaP—Hydroxyapatite (HA) and ß-tricalcium phosphate (β-TCP); (**B**) everage ALP activity shown by each MSC on both CaPs—Adipose tissue (ASCs), dental pulp (DPSCs), and bone marrow (BM-MSCs); (**C**) the average increase in ALP activity (in percentage) presented by each MSC on both CaPs—Adipose tissue (ASCs), dental pulp (DPSCs), and bone marrow (BM-MSCs); and (**D**) increase in ALP activity (in percentage) presented by each construct—Hydroxyapatite (HA), ß-tricalcium phosphate (β-TCP), adipose tissue (ASCs), dental pulp (DPSCs), and bone marrow (BM-MSCs). All data are shown as mean ± standard deviation. Significance level: * *p* ≤ 0.05, ** *p* ≤ 0.01, *** *p* ≤ 0.001.

Figure 6A shows a significant differential ALP activity at both 24 h and 7 days, which was higher on HA (*t* = 24 h, β-TCP = 0.4249, HA = 0.6213, *p* = 0.0014; *t* = 7 days, β-TCP = 0.6201, HA = 0.9517, *p* = 0.0002).

According to cell localisation (Figure 6B), each localisation showed a significant increase in ALP activity (ASCs, t_1_ = 0.515, t_2_ = 0.9060, *p* = 0.0007; BM-MSCs, t_1_ = 0.6293, t_2_ = 0.8686, *p* = 0.0358; DPSCs, t_1_ = 0.417, t_2_ = 0.7147, *p* = 0.0002). The initial differences between locations disappeared at the final time point (*t* = 24 h, ASCs = 0.5150, BM-MSCs = 0.6293, DPSCs = 0.4170, *p*_ANOVA_ = 0.0306, *p*_DPSC-ASC_ = 0.2790, *p*_DPSC-BM-MSC_ = 0.0277, *p*_ASC-BM-MSC_ > 0.9999; *t* = 7 days, ASCs = 0.9016, BM-MSCs = 0.8636, DPSCs = 0.7147, *p*_ANOVA_ = 0.2394, *p*_DPSC-ASC_ = 0.3416, *p*_DPSC-BM-MSC_ = 0.5712, *p*_ASC-BM-MSC_ > 0.9999).

Finally, the increase in ALP activity (in percentage) was calculated. DPSCs presented a bigger, although not significant, increase in ALP activity than the other sources (ASCs = 62.58%, BM-MSCs = 40.15%, DPSCs = 72.51%, *p*_ANOVA_ = 0.1479, *p*_DPSC-ASC_ > 0.9999, *p*_DPSC-BM-MSC_ = 0.1969, *p*_ASC-BM-MSC_ = 0.4091; Figure 6C). Regarding biomaterial–cell combinations, DPSC + HA increased the most, although no significant differences were obtained (ASCs + HA = 98.60%, BM-MSCs + HA = 43.46%, DPSCs + HA = 132.20%, ASCs + β-TCP = 78.38%, BM-MSCs + β-TCP = 112.20%, DPSCs + β-TCP = 56.44%, *p*_ANOVA_ = 0.2425; Figure 6D).

## 4. Discussion

In the bone regeneration context, therapies based on the use of osteoprogenitor cells have shown promise. However, the full therapeutic potential of these techniques has not yet been achieved. A full understanding of how different biological aspects influence the osteogenic potential is required. The anatomical localisation of the cells used is among those characteristics. To our knowledge, this is the first comparative work that has analysed a possible osteogenic commitment depending on the anatomic localisation of non-commercial human MSCs from bone marrow, adipose tissue, and dental pulp seeded both on a plastic surface and CaPs (β-TCP and HA).

The starting point of this work was the analysis of the results obtained in a previous iTRAQ analysis, where MSCs from subchondral bone and cartilage were compared in an osteoarthritis (OA) study. OA is a condition characterised by excessive bone growth, with the main affected tissues being subchondral bone and cartilage. Specifically, the results obtained after comparing these locations in healthy individuals were analysed in a free-hypothesis context. Fifty proteins were identified (Table 2). Five of those 50 proteins have been communicated as being related to the osteogenic process. FLNA, HSPA5/GRP78, and PALLD were up-regulated in subchondral bone, and their expression has been correlated with osteoblast differentiation, as they contribute to the stabilisation of cytoskeleton, which is necessary for the osteogenesis and regulation of protein folding and calcium flux [34,35,36,37]. In contrast, DSTN and IBP3—inhibitors of osteoblast differentiation [38,39]—were down-regulated in MSCs isolated from the subchondral bone. Considering this background, MScs from subchondral bone display a more established commitment to osteogenesis, compared to MSCs from cartilage. This supports the hypothesis that anatomical proximity to bone has a direct effect on MSC phenotype, in terms of increased osteogenic commitment. Further studies are required to clarify whether this commitment is due to cell location or cell inherent properties.

**Figure A1:**
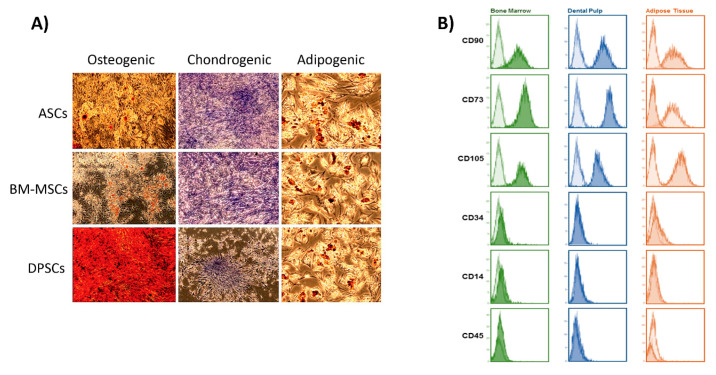
Characterisation of MSCs: (**A**) lineage-specific staining to confirm the correct differentiation into osteogenic, chondrogenic, and adipogenic lineages. Microscopy images show that all MSCs present the ability to differentiate into the 3 lineages; (**B**) flow cytometry results after analysing the surface marker profile of each MSC.

To confirm this hypothesis, we analysed the biological behaviour of MSCs isolated from locations with different proximity to bone (adipose tissue, bone marrow, and dental pulp) on different surfaces. All cells used in this work met the minimal criteria to be defined as MSCs (Figure A1) [31].

On plastic surfaces, proliferative ability and osteogenic potential were studied. Evaluating cell proliferation is important for cell-based therapies, as it has been communicated that stem cell therapy failures are likely to be due to a massive cell death occurring after cell transplantation [40]. Our results established that DPSCs have the highest proliferation rate (Figure 1A), in line with previous studies [19,24]. Furthermore, it was observed that DPSCs presented a smaller size. As DPSCs have a higher proliferation rate, this smaller size is likely a consequence, due to the indirect relationship between proliferation and cell size [41]. Alizarin Red staining is commonly used as an osteogenic differentiation indicator, as mineralized nodules are red-coloured. Microscopy images show that DPSCs exhibited the most intense staining, with its quantification evidencing significant differences (Figure 1B). These results indicate that DPSCs presented the most osteogenic capacity in vitro, followed by BM-MSCs and ASCs as the least osteogenic. This staining pattern confirms and extends the results obtained by Tamaki et al. [19]. Furthermore, it has been described that DPSCs likely have an advantage for osteogenic differentiation over other MSCs [22], as DPSCs only differentiate into osteoblasts at high passages [42].

Once the constructs were generated, we studied their cell viability, colonisation ability, and osteogenic capacity. The viability test showed that β-TCP was more cell-friendly than HA (Figure 2A,C). Both β-TCP and HA are biomaterials commonly used in bone tissue engineering and dentistry to treat bone defects. Implant surface quality is a major factor in biocompatibility. When the surface of the implanted biomaterial is exposed to tissue fluids, an initial interaction occurs between the living bone and tissue and the implant surface. In this sense, the use of materials filled with tricalcium phosphate appears promising, following the observation that more living cells are in this material [43]. Regarding MSCs, the viability test was favourable for DPSCs at 24 h and 4 days, compared to ASCs or BM-MSCs; however, these differences were not significant at 7 days (Figure 2B).

SEM images (Figure 3 and Figure 4) were coherent, as only DPSCs developed a cell layer on HA; whereas, on β-TCP, all three source-derived MSCs achieved this. CaPs are porous, so pore colonisation by the cells is required for the optimal colonisation of the CaP. Specific images of the pores existing in both CaPs prove the pores were successfully colonised by all MSCs after 24 h (Figure 5). Furthermore, cytotoxicity was not observed in the studied samples. Remarkably, DPSCs showed the greatest proliferation ability on both plastic and bioceramic surfaces. This feature provides an advantage for bone regeneration, as high MSCs density enhances osteogenic differentiation [44]. These results, together with those on plastic surfaces and previous studies, highlight the features of DPSCs for dental and bone regeneration applications [21,45].

The interplay of the tissue engineering triad (cells, signalling molecules, and scaffolds) is essential for recapitulation of the biological events of tissue regeneration. These elements have been used, either separately or in combination, for the reconstitution of the pulp–dentin complex and bone defects. In the literature, it is established that the mechanical properties of the scaffold influence cell processes such as proliferation and differentiation [46]. Recent data have implied that β-TCP is a bioactive and biocompatible material, capable of enhancing the proliferation, migration, and adhesion of DPSCs. Moreover, recent data have been conclusive about the higher levels of osteogenic and odontogenic differentiation markers related to DPSCs, such as COLI, DSPP, OC, RUNX2, and DMP-1. Our results suggest, in accordance with the literature, that DPSCs may be a valuable tool in the context of dental and bone regeneration [47].

**Figure A2:**
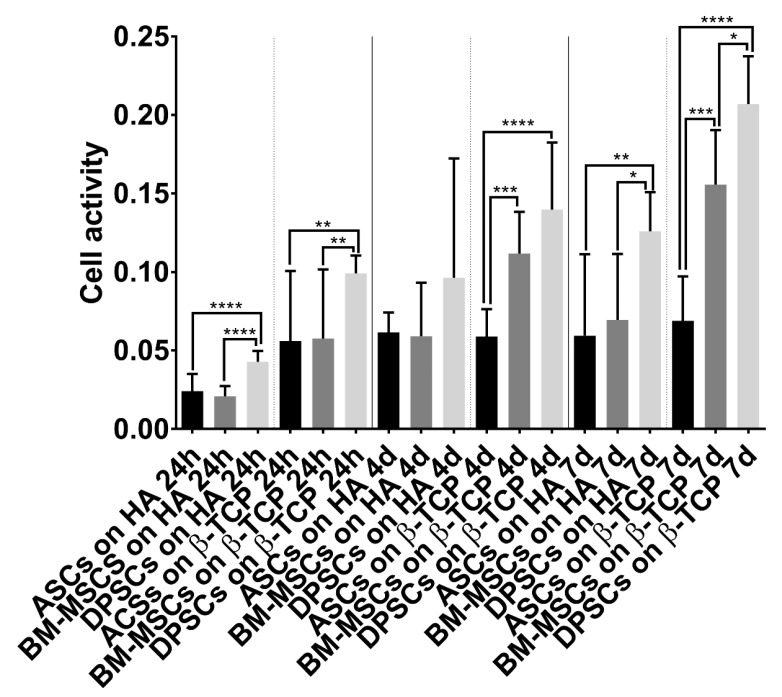
Cell activity during induced osteogenesis. Alamar Blue test results obtained by MSC–CaP combinations in osteogenic medium at the following time points: 24 h, 4 days, and 7 days. Data are shown as mean ± standard deviation. Significance level: * *p* ≤ 0.05, ** *p* ≤ 0.01, *** *p* ≤ 0.001, **** *p* ≤ 0.0001.

Regarding osteogenic potential, the ALP activity test results (Figure 6A) mismatched with the viability results (Figure 2A): while β-TCP appeared to be the best in terms of viability, HA obtained the highest values in ALP activity. These results indicate that a combination of materials may be more effective. Referring to cell sources, ASCs and BM-MSCs presented similar absolute values, which were slightly superior to that of DPSCs, but not significant (Figure 6B). Interestingly, this pattern in ALP activity was obtained in recent studies comparing multisource-derived MSCs seeded on plastic surfaces, although authors communicated significant differences [23,24]. The absence of significance in our results could be due to the osteoinductive properties that both CaPs present, which the plastic surface lacked [48,49]. As DPSCs presented the highest metabolic activity during osteogenesis on both CaPs (Figure A2), we consider it conceivable that CaPs can enhance osteogenesis preferentially in DPSCs over other MSCs.

## 5. Conclusions

Our results indicate DPSCs as the ideal cell for bone regeneration scenarios. Within bone regeneration, DPSCs are potentially beneficial in periodontal regeneration. Supporting this, a Phase 3 clinical study using DPSCs for alveolar cleft lip and palate repair has recently been initiated (ClinicalTrials.gov Identifier: NCT03766217), and promising results regarding the use of DPSCs for periodontal regeneration have been published [50]. Moreover, a combination of the best viability obtained with β-TCP and the enhanced osteogenic capacity of HA may be appropriate. Future studies are necessary to obtain the best combination of cells and biomaterials, together with other signalling enhancers or inhibitors and different proteins that have been demonstrated in the field of dentistry and bone regeneration [51].

## Figures and Tables

**Table 1:** Main characteristics of healthy donors.

Donor	Cell Type	Sex	Age
Donor 1	BM-MSC	F	57
Donor 2	BM-MSC	M	56
Donor 3	BM-MSC	M	55
Donor 4	ASC	F	53
Donor 5	ASC	F	56
Donor 6	ASC	M	50
Donor 7	DPSC	F	44
Donor 8	DPSC	M	58
Donor 9	DPSC	M	59

BM-MSC: Bone marrow mesenchymal stem cell; ASC: Adipose-derived mesenchymal stem cell; DPSC: Dental pulp mesenchymal stem cell.

**Table 2:** Differentially expressed proteins identified in MSC isolated from subchondral bone, compared to that from cartilage.

Uniprot ID	Gene	Protein	Peptides	Fold Change	*p*-Value
AMPN_HUMAN	*ANPEP*	Aminopeptidase N	27	11.27	0.0001
**PALLD_HUMAN**	***PALLD***	**Palladin**	**4**	**4.6989**	**0.0145**
K2C1_HUMAN	*KRT1*	Keratin, type II cytoskeletal 1	11	4.6352	0.0164
TPM1_HUMAN	*TPM1*	Tropomyosin alpha-1 chain	13	4.3652	0.0173
TAGL_HUMAN	*TAGLN*	Transgelin	13	4.2855	0.0001
K1C10_HUMAN	*KRT10*	Keratin, type I cytoskeletal 10	8	3.5645	0.0085
FRIH_HUMAN	*FTH1*	Ferritin heavy chain	3	3.4356	0.034
DHB4_HUMAN	*HSD17B4*	Peroxisomal multifunctional enzyme type 2	5	3.4041	0.0078
VIME_HUMAN	*VIM*	Vimentin	235	3.3420	0.0291
SQRD_HUMAN	*SQRDL*	Sulphide:quinone oxidoreductase, mitochondrial	10	2.9923	0.0030
PDIA1_HUMAN	*P4HB*	Protein disulphide-isomerase	48	2.7290	0.0028
KPYM_HUMAN	*PKM2*	Pyruvate kinase isozymes M1/M2	69	2.6062	0.0123
ATPB_HUMAN	*ATP5B*	ATP synthase subunit beta, mitochondrial	21	2.5351	0.0118
**GRP78_HUMAN**	***HSPA5***	**78 kDa glucose-regulated protein**	**53**	**2.5119**	**0.0001**
GLCM_HUMAN	*GBA*	Glucosylceramidase	4	2.4210	0.0472
DPYL3_HUMAN	*DPYSL3*	Dihydropyrimidinase-related protein 3	14	2.3768	0.0232
SPTB2_HUMAN	*SPTBN1*	Spectrin beta chain, brain 1	31	2.0512	0.0251
GDIR1_HUMAN	*ARHGDIA*	Rho GDP-dissociation inhibitor 1	8	2.0137	0.0105
ACADV_HUMAN	*ACADVL*	Very long-chain specific acyl-CoA dehydrogenase, mitochondrial	7	1.8707	0.0486
ATPA_HUMAN	*ATP5A1*	ATP synthase subunit alpha, mitochondrial	24	1.8535	0.0070
MYL6_HUMAN	*MYL6*	Myosin light polypeptide 6	16	1.7865	0.0487
LMO7_HUMAN	*LMO7*	LIM domain only protein 7	11	1.6144	0.0436
5NTD_HUMAN	*NT5E*	5′-nucleotidase	22	1.5704	0.0440
**FLNA_HUMAN**	***FLNA***	**Filamin-A**	**126**	**1.5276**	**0.0036**
HS90A_HUMAN	*HSP90AA1*	Heat shock protein HSP 90-alpha	29	1.5276	0.0351
H32_HUMAN	*HIST2H3A*	Histone H3.2	7	0.8872	0.0298
H2AJ_HUMAN	*H2AFJ*	Histone H2A.J	7	0.7798	0.0365
PDLI7_HUMAN	*PDLIM7*	PDZ and LIM domain protein 7	1	0.7311	0.0042
ALDR_HUMAN	*AKR1B1*	Aldose reductase	5	0.6918	0.0237
FLNC_HUMAN	*FLNC*	Filamin-C	14	0.6310	0.0145
MVP_HUMAN	*MVP*	Major vault protein	23	0.5649	0.0078
VINC_HUMAN	*VCL*	Vinculin	23	0.5546	0.0245
RS15A_HUMAN	*RPS15A*	40S ribosomal protein S15a	4	0.5248	0.0221
SFPQ_HUMAN	*SFPQ*	Splicing factor, proline- and glutamine-rich	3	0.4966	0.0205
PLIN3_HUMAN	*PLIN3*	Perilipin-3	14	0.4875	0.0418
NNMT_HUMAN	*NNMT*	Nicotinamide N-methyltransferase	6	0.4742	0.0400
1433Z_HUMAN	*YWHAZ*	14-3-3 protein zeta/delta	14	0.4699	0.0217
PRDX1_HUMAN	*PRDX1*	Peroxiredoxin-1	21	0.4169	0.0199
ANX11_HUMAN	*ANXA11*	Annexin A11	9	0.3733	0.0014
G6PD_HUMAN	*G6PD*	Glucose-6-phosphate 1-dehydrogenase	15	0.3664	0.0006
HYEP_HUMAN	*EPHX1*	Epoxide hydrolase 1	7	0.3467	0.0069
6PGD_HUMAN	*PGD*	6-phosphogluconate dehydrogenase, decarboxylating	10	0.2911	0.0040
CALU_HUMAN	*CALU*	Calumenin	14	0.2655	0.0057
NQO1_HUMAN	*NQO1*	NAD(P)H dehydrogenase [quinone] 1	11	0.2512	0.0153
TKT_HUMAN	*TKT*	Transketolase	21	0.1486	0.0051
ANXA5_HUMAN	*ANXA5*	Annexin A5	38	0.1294	0.0002
ENOA_HUMAN	*ENO1*	Alpha-enolase	54	0.1000	0.0419
ANXA2_HUMAN	*ANXA2*	Annexin A2	74	0.0871	0.0019
**IBP3_HUMAN**	***IGFBP3***	**Insulin-like growth factor-binding protein 3**	**5**	**0.0802**	**0.0424**
**DEST_HUMAN**	***DSTN***	**Destrin**	**8**	**0.0614**	**0.0245**

Note: Proteins are listed, according to fold change, in decreasing order. In bold, proteins previously related to the osteogenic process.

## Data Availability

The study reports no data.

## References

[1] Riester O., Borgolte M., Csuk R., Deigner H.-P. (2020). Challenges in Bone Tissue Regeneration: Stem Cell Therapy, Biofunctionality and Antimicrobial Properties of Novel Materials and Its Evolution. Int. J. Mol. Sci..

[2] Einhorn T.A. (1998). The Cell and Molecular Biology of Fracture Healing. Clin. Orthop. Relat. Res..

[3] Giannoudis P.V., Calori G.M., Begue T., Schmidmaier G. (2013). Bone regeneration strategies: Current trends but what the future holds?. Injury.

[4] Qin W., Chen J.-Y., Guo J., Ma T., Weir M.D., Guo D., Shu Y., Lin Z.-M., Schneider A., Xu H.H.K. (2018). Novel Calcium Phosphate Cement with Metformin-Loaded Chitosan for Odontogenic Differentiation of Human Dental Pulp Cells. Stem Cells Int..

[5] Johnson A.J.W., Herschler B.A. (2011). A review of the mechanical behavior of CaP and CaP/polymer composites for applications in bone replacement and repair. Acta Biomater..

[6] Descamps M., Boilet L., Moreau G., Tricoteaux A., Lu J., Leriche A., Lardot V., Cambier F. (2013). Processing and properties of biphasic calcium phosphates bioceramics obtained by pressureless sintering and hot isostatic pressing. J. Eur. Ceram. Soc..

[7] Da Silva Meirelles L., Chagastelles P.C., Nardi N.B. (2006). Mesenchymal stem cells reside in virtually all post-natal organs and tissues. J. Cell Sci..

[8] Pittenger M.F., Mackay A.M., Beck S.C., Jaiswal R.K., Douglas R., Mosca J.D., Moorman M.A., Simonetti D.W., Craig S., Marshak D.R. (1999). Multilineage potential of adult human mesenchymal stem cells. Science.

[9] Yoo K.H., Jang I.K., Lee M.W., Kim H.E., Yang M.S., Eom Y., Lee J.E., Kim Y.J., Yang S.K., Jung H.L. (2009). Comparison of immunomodulatory properties of mesenchymal stem cells derived from adult human tissues. Cell. Immunol..

[10] Sun C., Zhang K., Yue J., Meng S., Zhang X. (2021). Deconstructing transcriptional variations and their effects on immunomodulatory function among human mesenchymal stromal cells. Stem Cell Res. Ther..

[11] Stubbendorff M., Deuse T., Hua X., Phan T.T., Bieback K., Atkinson K., Eiermann T.H., Velden J., Schröder C., Reichenspurner H. (2013). Immunological Properties of Extraembryonic Human Mesenchymal Stromal Cells Derived from Gestational Tissue. Stem Cells Dev..

[12] Escacena N., Quesada-Hernández E., Capilla-Gonzalez V., Soria B., Hmadcha A. (2015). Bottlenecks in the Efficient Use of Advanced Therapy Medicinal Products Based on Mesenchymal Stromal Cells. Stem Cells Int..

[13] Friedenstein A.J., Gorskaja J.F., Kulagina N.N. (1976). Fibroblast precursors in normal and irradiated mouse hematopoietic organs. Exp. Hematol..

[14] Zhu Y., Liu T., Song K., Fan X., Ma X., Cui Z. (2008). Adipose-derived stem cell: A better stem cell than BMSC. Cell Biochem. Funct..

[15] Xia L., Lin K., Jiang X., Fang B., Xu Y., Liu J., Zeng D., Zhang M., Zhang X., Chang J. (2014). Effect of nano-structured bioceramic surface on osteogenic differentiation of adipose derived stem cells. Biomaterials.

[16] Spath L., Rotilio V., Alessandrini M., Gambara G., De Angelis L., Mancini M., Mitsiadis T.A., Vivarelli E., Naro F., Filippini A. (2009). Explant-derived human dental pulp stem cells enhance differentiation and proliferation potentials. J. Cell. Mol. Med..

[17] Yu J., He H., Tang C., Zhang G., Li Y., Wang R., Shi J., Jin Y. (2010). Differentiation potential of STRO-1+ dental pulp stem cells changes during cell passaging. BMC Cell Biol..

[18] Kichenbrand C., Velot E., Menu P., Moby V. (2019). Dental Pulp Stem Cell-Derived Conditioned Medium: An Attractive Alternative for Regenerative Therapy. Tissue Eng. Part B Rev..

[19] Tamaki Y., Nakahara T., Ishikawa H., Sato S. (2013). In vitro analysis of mesenchymal stem cells derived from human teeth and bone marrow. Odontology.

[20] Li C., Wu X., Tong J., Yang X., Zhao J., Zheng Q., Zhao G., Ma Z. (2015). Comparative analysis of human mesenchymal stem cells from bone marrow and adipose tissue under xeno-free conditions for cell therapy. Stem Cell Res. Ther..

[21] Lobo S.E., Glickman R., da Silva W.N., Arinzeh T.L., Kerkis I. (2015). Response of stem cells from different origins to biphasic calcium phosphate bioceramics. Cell Tissue Res..

[22] Ren H., Sang Y., Zhang F., Liu Z., Qi N., Chen Y. (2016). Comparative Analysis of Human Mesenchymal Stem Cells from Umbilical Cord, Dental Pulp, and Menstrual Blood as Sources for Cell Therapy. Stem Cells Int..

[23] D’Alimonte I., Mastrangelo F., Giuliani P., Pierdomenico L., Marchisio M., Zuccarini M., Di Iorio P., Quaresima R., Caciagli F., Ciccarelli R. (2017). Osteogenic Differentiation of Mesenchymal Stromal Cells: A Comparative Analysis Between Human Subcutaneous Adipose Tissue and Dental Pulp. Stem Cells Dev..

[24] Zhang Y., Xing Y., Jia L., Ji Y., Zhao B., Wen Y., Xu X. (2018). An In Vitro Comparative Study of Multisource Derived Human Mesenchymal Stem Cells for Bone Tissue Engineering. Stem Cells Dev..

[25] Hornez J.C., Chai F., Monchau F., Blanchemain N., Descamps M., Hildebrand H.F. (2007). Biological and physico-chemical assessment of hydroxyapatite (HA) with different porosity. Biomol. Eng..

[26] Descamps M., Duhoo T., Monchau F., Lu J., Hardouin P., Hornez J.C., Leriche A. (2008). Manufacture of macroporous β-tricalcium phosphate bioceramics. J. Eur. Ceram. Soc..

[27] Yang X.-F., He X., He J., Zhang L.-H., Su X.-J., Dong Z.-Y., Xu Y.-J., Li Y., Li Y.-L. (2011). High efficient isolation and systematic identification of human adipose-derived mesenchymal stem cells. J. Biomed. Sci..

[28] Huang G.T.J., Sonoyama W., Chen J., Park S.H. (2006). In vitro characterization of human dental pulp cells: Various isolation methods and culturing environments. Cell Tissue Res..

[29] Gudleviciene Z., Kundrotas G., Liudkeviciene R., Rascon J., Jurga M. (2015). Quick and effective method of bone marrow mesenchymal stem cell extraction. Open Med..

[30] Alkhalil M., Smajilagić A., Redžić A. (2015). Human dental pulp mesenchymal stem cells isolation and osteoblast differentiation. Med. Glas..

[31] Dominici M., Le Blanc K., Mueller I., Slaper-Cortenbach I., Marini F., Krause D.S., Deans R.J., Keating A., Prockop D.J., Horwitz E.M. (2006). Minimal criteria for defining multipotent mesenchymal stromal cells. The International Society for Cellular Therapy position statement. Cytotherapy.

[32] Tornero-Esteban P., Peralta-Sastre A., Herranz E., Rodríguez-Rodríguez L., Mucientes A., Abásolo L., Marco F., Fernández-Gutiérrez B., Lamas J.R. (2015). Altered Expression of Wnt Signaling Pathway Components in Osteogenesis of Mesenchymal Stem Cells in Osteoarthritis Patients. PLoS ONE.

[33] Golub E.E., Boesze-Battaglia K. (2007). The role of alkaline phosphatase in mineralization. Curr. Opin. Orthop..

[34] Wall M.E., Rachlin A., Otey C.A., Loboa E.G. (2007). Human adipose-derived adult stem cells upregulate palladin during osteogenesis and in response to cyclic tensile strain. Am. J. Physiol. Cell Physiol..

[35] Yu H., Tay C.Y., Leong W.S., Tan S.C.W., Liao K., Tan L.P. (2010). Mechanical behavior of human mesenchymal stem cells during adipogenic and osteogenic differentiation. Biochem. Biophys. Res. Commun..

[36] Tan J., Zhou L., Xue P., An Y., Luo L., Zhang R., Wu G., Wang Y., Zhu H., Wang Q. (2016). Tumor Necrosis Factor-α Attenuates the Osteogenic Differentiation Capacity of Periodontal Ligament Stem Cells by Activating PERK Signaling. J. Periodontol..

[37] Corsetti G., Romano C., Stacchiotti A., Pasini E., Dioguardi F.S. (2017). Endoplasmic Reticulum Stress and Apoptosis Triggered by Sub-Chronic Lead Exposure in Mice Spleen: A Histopathological Study. Biol. Trace Elem. Res..

[38] Chen L., Shi K., Frary C.E., Ditzel N., Hu H., Qiu W., Kassem M. (2015). Inhibiting actin depolymerization enhances osteoblast differentiation and bone formation in human stromal stem cells. Stem Cell Res..

[39] Eguchi K., Akiba Y., Akiba N., Nagasawa M., Cooper L.F., Uoshima K. (2018). Insulin-like growth factor binding Protein-3 suppresses osteoblast differentiation via bone morphogenetic protein-2. Biochem. Biophys. Res. Commun..

[40] Chacko S.M., Ahmed S., Selvendiran K., Kuppusamy M.L., Khan M., Kuppusamy P. (2010). Hypoxic preconditioning induces the expression of prosurvival and proangiogenic markers in mesenchymal stem cells. Am. J. Physiol. Physiol..

[41] Tzur A., Kafri R., LeBleu V.S., Lahav G., Kirschner M.W. (2009). Cell Growth and Size Homeostasis in Proliferating Animal Cells. Science.

[42] Yu J., Wang Y., Deng Z., Tang L., Li Y., Shi J., Jin Y. (2007). Odontogenic capability: Bone marrow stromal stem cells versus dental pulp stem cells. Biol. Cell.

[43] Kozakiewicz M., Wach T. (2020). New Oral Surgery Materials for Bone Reconstruction—A Comparison of Five Bone Substitute Materials for Dentoalveolar Augmentation. Materials.

[44] Wang X., Song W., Kawazoe N., Chen G. (2013). The osteogenic differentiation of mesenchymal stem cells by controlled cell-cell interaction on micropatterned surfaces. J. Biomed. Mater. Res. Part A.

[45] Wongsupa N., Nuntanaranont T., Kamolmattayakul S., Thuaksuban N. (2017). Biological characteristic effects of human dental pulp stem cells on poly-ε-caprolactone-biphasic calcium phosphate fabricated scaffolds using modified melt stretching and multilayer deposition. J. Mater. Sci. Mater. Med..

[46] Dey K., Roca E., Ramorino G., Sartore L. (2020). Progress in the mechanical modulation of cell functions in tissue engineering. Biomater. Sci..

[47] Sabbagh J., Ghassibe-Sabbagh M., Fayyad-Kazan M., Al-Nemer F., Fahed J.C., Berberi A., Badran B. (2020). Differences in osteogenic and odontogenic differentiation potential of DPSCs and SHED. J. Dent..

[48] Thorpe A.A., Creasey S., Sammon C., Le Maitre C.L. (2016). Hydroxyapatite nanoparticle injectable hydrogel scaffold to support osteogenic differentiation of human mesenchymal stem cells. Eur. Cell. Mater..

[49] Tsukanaka M., Fujibayashi S., Otsuki B., Takemoto M., Matsuda S. (2015). Osteoinductive potential of highly purified porous β-TCP in mice. J. Mater. Sci. Mater. Med..

[50] Ferrarotti F., Romano F., Gamba M.N., Quirico A., Giraudi M., Audagna M., Aimetti M. (2018). Human intrabony defect regeneration with micrografts containing dental pulp stem cells: A randomized controlled clinical trial. J. Clin. Periodontol..

[51] Fischer N.G., Münchow E.A., Tamerler C., Bottino M.C., Aparicio C. (2020). Harnessing biomolecules for bioinspired dental biomaterials. J. Mater. Chem. B.

